# Successful management of octreotide-insensitive thyrotropin-secreting pituitary adenoma with bromocriptine and surgery: A case report and literature review

**DOI:** 10.1097/MD.0000000000008017

**Published:** 2017-09-08

**Authors:** Chengxian Yang, Huanwen Wu, Jing Wang, Mingming Hu, Xiaoping Xing, Xinjie Bao, Renzhi Wang

**Affiliations:** a Department of Neurosurgery; b Department of Pathology; c Department of Endocrinology, Key Laboratory of Endocrinology of National Health And Family Planning Commission, Peking Union Medical College Hospital, Chinese Academy of Medical Sciences & Peking Union Medical College, No. 1 Shuaifuyuan Hutong of Dongcheng District, Beijing, China.

**Keywords:** bromocriptine, case report, dopamine agonist, medical treatment, thyrotropin-secreting pituitary adenoma

## Abstract

**Rationale:**

Case reports concerning the value of dopamine agonists in the treatment of patients with thyrotropin-secreting pituitary adenoma (TSHoma) are limited. Herein, we present a rare case of octreotide-insensitive TSHoma responding to bromocriptine therapy.

**Patient concerns:**

A 45-year-old Chinese man was admitted to Peking Union Medical College Hospital with marked clinical manifestations of hyperthyroidism.

**Diagnoses:**

Thyroid function tests demonstrated elevated concentrations of free thyroid hormones in the presence of normal thyrotropin. Magnetic resonance imaging findings showed a pituitary microadenoma on the right side of the sellar region. Based on characteristic endocrine results and neuroimaging findings, the patient was diagnosed with TSHoma.

**Interventions:**

Most patients with TSHomas are significantly responsive to somatostatin analog treatment. However, our patient was orally administered with bromocriptine to normalize thyroid function as assessed by suppression tests conducted prior to surgery. A transsphenoidal surgery was performed by an experienced neurosurgeon for tumor removal.

**Outcomes:**

The pituitary lesion was totally resected. Following the operation, the results of thyroid function tests were immediately within reference limits. During the follow-up, there was no residual or recurrent tumor.

**Lessons:**

Attention should be paid to the role of dopamine agonists such as bromocriptine and cabergoline as adjuvant therapy for TSHomas that are insensitive to traditional medical treatment by somatostatin analogs.

## 1 Introduction

Thyrotropin-secreting pituitary adenoma (TSHoma) is a rare cause of hyperthyroidism, representing approximately 0.5% to 3% of all pituitary adenomas.^[1]^ The first case report of TSHoma, published in 1960, described a patient recovered from Grave's disease after pituitary radiotherapy.^[2]^ The epidemiological data of TSHoma is limited worldwide. According to national data from Sweden, the incidence of TSHoma is approximately 0.15 per million per year, with a tendency toward growth, and the prevalence is about 1 per million.^[3]^

The diagnosis of TSHoma remains a challenge despite advances in hormone assays and neuroimaging. It is characterized by high-level free thyroid hormones on a background of inappropriately normal or elevated thyrotropin (thyroid-stimulating hormone, TSH) concentrations. Magnetic resonance imaging (MRI) findings indicate that TSHoma generally initially presents as a macroadenoma with hypoenhanced features and a tendency toward invasiveness.^[4]^ Currently, the diagnosis of TSHoma depends primarily on endocrine results and the appearance of a lesion following pituitary imaging.^[5]^ The treatment of TSHoma consists of surgery, medical management, and radiotherapy. Transsphenoidal adenomectomy is the first-line treatment option. Medical management mainly includes primary treatment with somatostatin analogs (SSAs), such as octreotide acetate, and dopamine agonists, which can achieve euthyroidism and tumor shrinkage. Radiotherapy is commonly recommended for patients with surgical failure or contraindications.

In this case report and literature review, we describe the effectiveness and clinical value of dopamine agonists in TSHoma treatment, suggesting that this therapy warrants further investigation.

## 2 Case report

A 45-year-old Chinese man was admitted to Peking Union Medical College Hospital (PUMCH) in April 2016 because of progressively worsening palpitation and hand tremor during the previous 6 months together with 4 years of mild weakness, heat intolerance, increased perspiration, and growing appetite, which were previously spontaneously alleviated. His past and family histories were unremarkable, with no history of thyroidectomy, and findings from a general physical examination were also unremarkable. The patient did not seek medical services until February 2016. In the local hospital, the thyroid function tests demonstrated elevated levels of free thyroxine (FT4; 40.04 pmol/L; reference range, 12–22 pmol/L) and free triiodothyronine (FT3; 17.02 pmol/L; reference range, 3.1–6.8 pmol/L) in the presence of normal levels of TSH (4.15 mIU/L; reference range, 0.27–4.2 mIU/L). The results of a dynamic electrocardiogram demonstrated arrhythmias, including transient atrial tachycardia, paroxysmal atrial fibrillation, and ventricular and atrial premature beats. Thyroid ultrasound examination results indicated cystic nodules in both thyroid lobes and diffuse enlargement of the thyroid gland. Thyroid scintigraphy findings showed normal radionuclide uptake. Thus, the patient was initially diagnosed as having central hyperthyroidism and suspected TSHoma although a brain MRI was not conducted.

**Figure 1:**
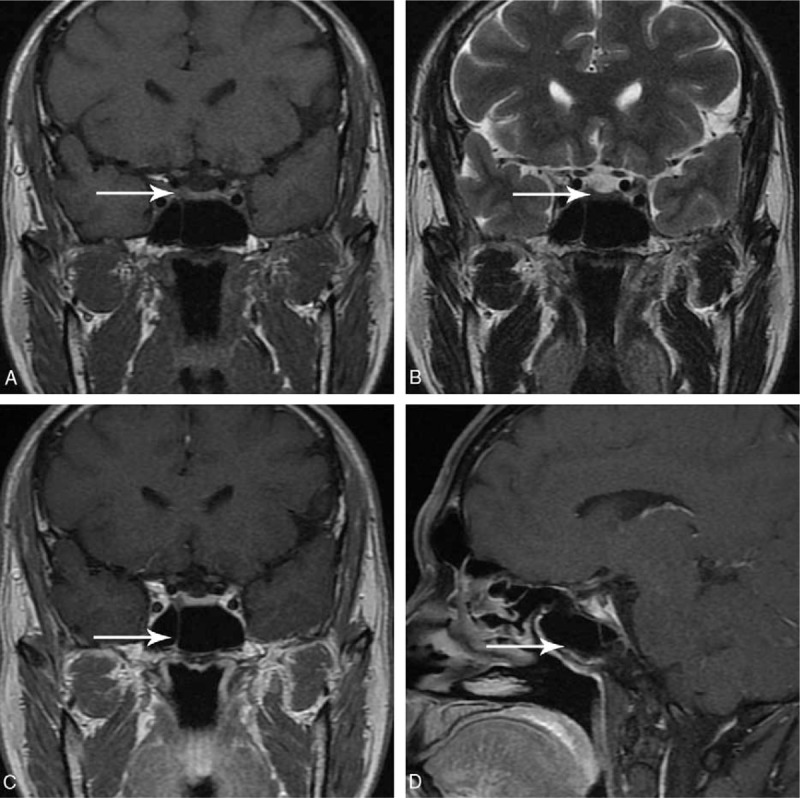
Preoperative MRI characteristics of the pituitary lesion: (A) coronal T1WI, (B) coronal T2WI, (C) coronal-enhanced T1WI, (D) sagittal-enhanced T1WI). MRI = magnetic resonance imaging, T1WI = T1-weighted image, T2WI = T2-weighted image.

**Figure 2:**
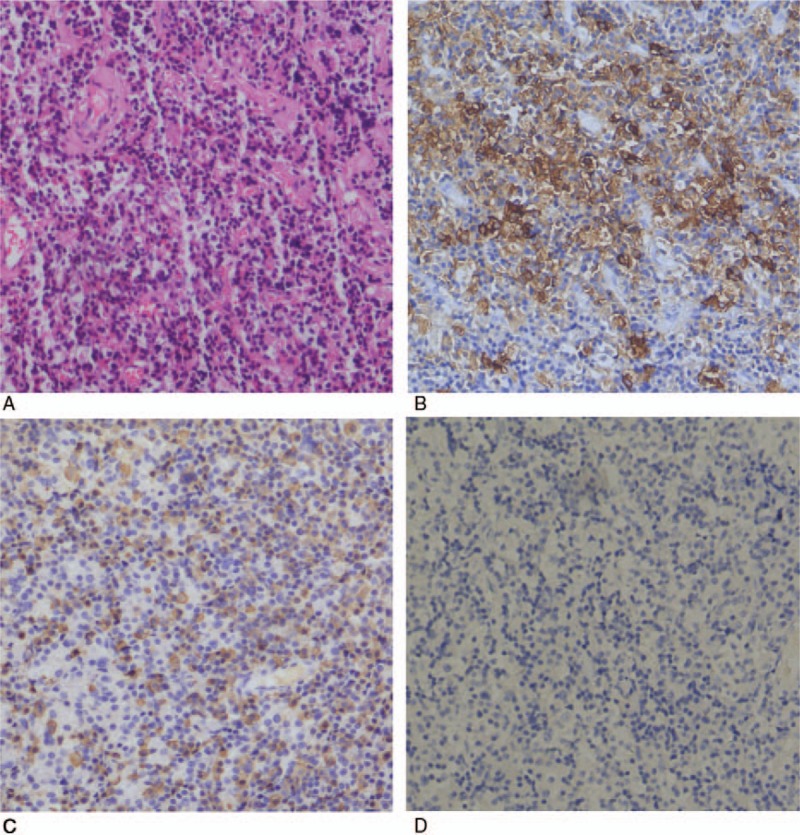
Histopathological and immunohistochemical characteristics of thyrotropin-secreting pituitary adenoma: (A) hematoxylin and eosin stain × 100, (B) immunohistochemical stain for TSH ×100, (C) immunohistochemical stain for GH ×100, (D) immunohistochemical stain for PRL × 100. GH = growth hormone, PRL = prolactin, TSH = thyroid stimulating hormone.

**Figure 3:**
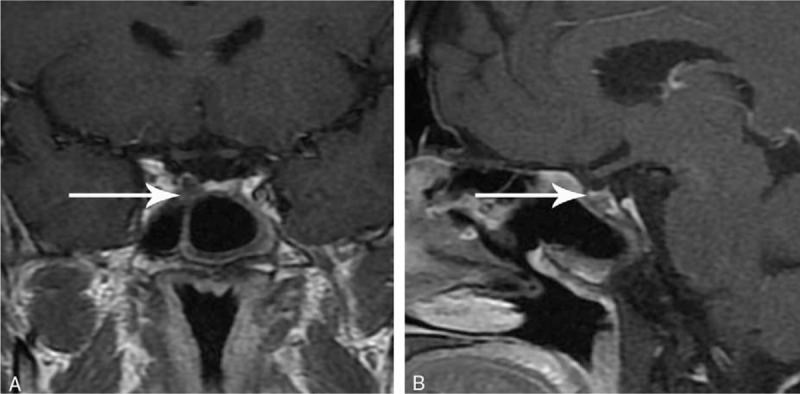
Postoperative MRI characteristics of the pituitary lesion: (A) coronal-enhanced T1WI, (B) sagittal-enhanced T1WI. MRI = magnetic resonance imaging, T1WI = T1-weighted image.

On his admission to PUMCH, further examinations were performed to confirm or reject the suspected TSHoma. Thyroid function tests were conducted in a biochemical laboratory to determine whether the previously reported FT3 and FT4 levels had been falsely elevated by the measurement methods used. However, our results still identified high FT3 and FT4 concentrations in the presence of unsuppressed TSH (Table 1). The serum sex hormone-binding globulin level was elevated (105.08 nmol/L; reference range, 18.3–54.1 nmol/L). Unfortunately, triiodothyronine (T3) suppression tests and thyrotropin-releasing hormone (TRH) stimulation tests were not available at PUMCH. The pituitary function was comprehensively evaluated and showed no co-secretion of growth hormone (GH) and prolactin (PRL) (Table 1). Mutations in the gene encoding thyroid hormone receptor β (THRB) were not identified. The ophthalmology testing demonstrated no visual disturbance. A contrast-enhanced dynamic MRI of the pituitary showed an intrasellar microadenoma on the right side of the region measuring 4 mm × 6 mm × 6 mm. The lesion signal was hypointense on T1-weighted imaging (T1WI), hypointense on T2-weighted imaging (T2WI), and hypoenhanced on contrast-enhanced T1WI (Fig. 1). Together, these results strongly supported a diagnosis of TSHoma. A TSH suppression test was performed following the administration of octreotide acetate (0.1 mg, subcutaneously, once; sampling after 0, 2, 4, 6, 8, 24, and 72 hours; TSH levels were measured), and a marked reduction (>50%) in TSH levels, which is considered characteristic of TSHoma, was not observed. Thus, an additional TSH suppression test was conducted with bromocriptine mesylate administration (2.5 mg, orally, once; sampling after 0, 2, 4, 6, and 8 hours; TSH levels were measured) and the results demonstrated a notable suppression (61.2%) of TSH levels (Table 2). Therefore, the diagnosis of TSHoma was confirmed based on clinical manifestations, laboratory results, pituitary MRI findings, and TSH suppression test results. Propranolol (10 mg, orally, 3 times a day) was administered to control arrhythmia and tachycardia. The patient was also administered bromocriptine (2.5 mg, orally, every 8 hours) for 10 days to normalize thyroid function prior to surgery. A change in tumor volume was not detected. Bromocriptine was then discontinued and replaced by octreotide (0.1 mg, intramuscularly, once a day) for 3 days, resulting in recurrence of hyperthyroidism and related symptoms. Considering the ineffectiveness of the preoperative octreotide therapy, bromocriptine was reinstated as before for another 3 days, leading to the rapid normalization of thyroid function. A transsphenoidal adenomectomy was then performed. During the procedure, the grayish white lesion was totally resected, and leakage of cerebrospinal fluid did not occur. The histology results indicated that the lesion was a pituitary adenoma, and most neoplastic cells were strongly immunopositive for TSH and GH, whereas all neoplastic cells were immunonegative for PRL (Fig. 2). On the first day after surgery, thyroid function tests demonstrated a significant reduction in TSH levels and normal levels of FT3 and FT4 (Table 1). The postoperative course was otherwise uneventful. The patient was discharged on the fourth day after surgery. The patient was recommended to continue taking propranolol (10 mg, 3 times a day) and to use hydrocortisone for 2 weeks (week 1: 20 mg, once a day; week 2: 10 mg, once a day). At the 3-month follow-up visit, the results of thyroid function tests were within reference limits (Table 1), and pituitary MRI showed a normal pituitary gland with no recurrent or residual tumor (Fig. 3). At the 6-month follow-up, there were no signs or symptoms of hyperthyroidism or mass effects in the sellar region of our patient.

**Table 1:**
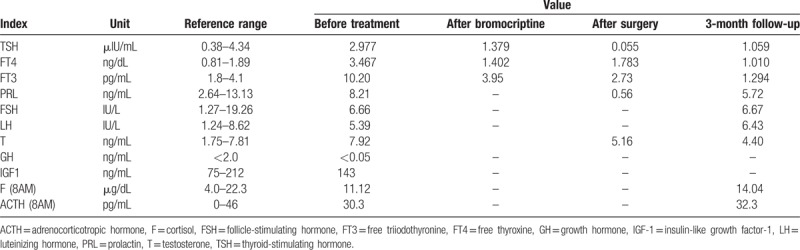
Data of hormone levels before and after treatment in PUMCH.

**Table 2:**
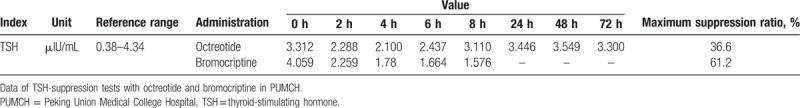
Results of TSH-suppression testing with administration of octreotide acetate and bromocriptine.

## 3 Discussion

Preoperative administration of octreotide is generally useful in TSHoma treatment. By contrast, the effectiveness of dopamine agonists in TSHoma treatment is limited; thus, this potential therapy may be easily overlooked. According to limited case reports and series^[6–13]^ (Table 3), dopamine agonists, including bromocriptine and cabergoline, have been shown to be useful in achieving euthyroidism and tumor shrinkage in a minority of patients (Table 4). Among these 11 patients, bromocriptine and octreotide suppression tests were administrated and well documented in only 3 patients (patients 1, 2, and 10). Only patient 2, who was from Japan, showed an octreotide-insensitive TSHoma that was responsive to bromocriptine, similar to the patient in our present case report. Thus, to the best of our knowledge, this is the first case report describing octreotide-insensitive TSHoma responding to bromocriptine mesylate in a Chinese individual.

**Table 3:**
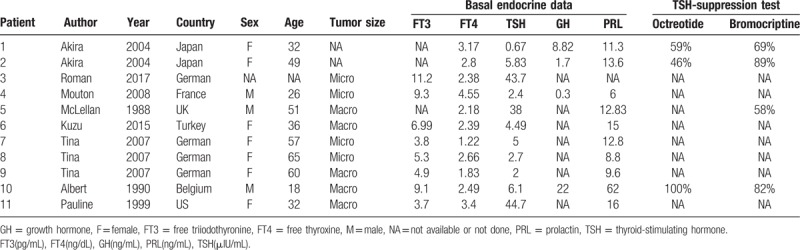
Summary of the characteristics of patients from the literature review.

**Table 4:**
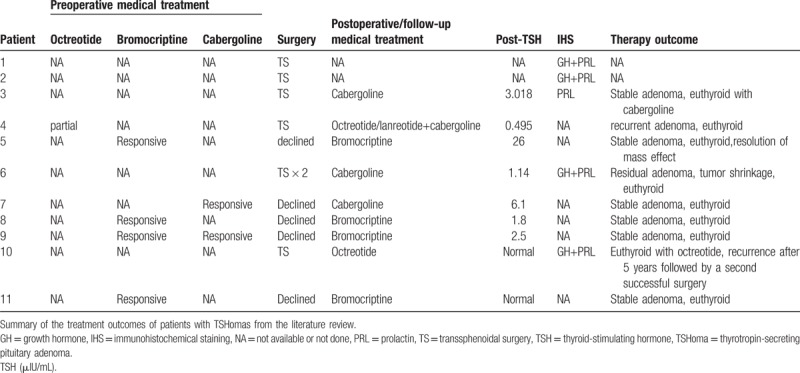
Summary of the treatment outcomes of patients with TSHomas.

Thyrotropin-secreting pituitary adenomas are commonly plurihormonal pituitary adenomas, co-secreting GH, PRL, or both. The immunohistochemical study results were available for 5 patients (patients 1, 2, 3, 6, and 10), and all 5 demonstrated immunopositive staining for PRL or PRL and GH. It has been argued that the co-existence of prolactinomas may contribute to dopamine agonist-induced tumor shrinkage and hormone control. However, Teramoto et al^[6]^ studied the mechanisms underlying the responses to medical treatments in TSHomas with different pathological characteristics and found no significant correlation between the medical response and co-secretion of GH and/or PRL. Previous studies have revealed gender-related difference of clinical presentations and treatment responses in prolactinomas, acromegaly, Cushing's disease and nonfunctioning pituitary adenomas.^[14]^ However, similar results were not reported in TSHomas. In our case, immunohistochemical results showed positive immunostaining of TSH and GH but negative immunostaining of PRL, indicating that the correlation between positive PRL staining and the responsiveness of dopamine agonists was not robust.

Good treatment response to dopamine agonists in pituitary adenomas is generally attributed to high expression levels of dopamine D2 receptors, such as in GH- and PRL-secreting adenoma. Bevan et al^[15]^ demonstrated that dopamine receptors were deficient in TSHomas that were irresponsive to bromocriptine. Wood et al^[16]^ revealed that dopamine agonists were of no value in TSHoma treatment despite the presence of dopamine D2 receptors. Furthermore, Wang et al^[17]^ studied the expression profile of dopamine D2 receptors in different subtypes of pituitary adenomas using immunohistochemical staining and western blotting and detected high dopamine D2 receptor expression levels in 60% of the tissues obtained from TSHomas. However, the rate of patients with TSHoma responding to dopamine agonist therapy is extremely low. Therefore, there is no confirmed link between the effectiveness of dopamine agonist treatment and the expression of dopamine D2 receptors in TSHoma. The mechanism for the dopamine agonist therapeutic effect in TSHoma awaits future research.

The purposes of preoperative medical treatment for TSHomas include tumor shrinkage and hormone normalization. With surgical advances in recent years, surgical outcomes of TSHomas are good regardless of tumor size.^[18]^ The rate of postoperative biochemical remission is 70% to 90%.^[7,18,19]^ In the past, a diagnosis of TSHoma was often delayed, resulting in an invasive macroadenoma, which complicates the surgical procedure. The preoperative MRI findings in our patient showed a microadenoma without mass effect in the sellar region, suggesting an early diagnosis in this case, significantly reducing the difficulty of the operation. Uncontrolled hyperthyroidism-related symptoms increase anesthetic and surgical risks. Our patient presented with worsening palpitation and arrhythmia. The preoperative use of bromocriptine and propranolol effectively alleviated the cardiac symptoms in our patient. Thus, appropriate preoperative preparations should be aimed at reducing surgical difficulty and risk.

Although 11 patients displaying great responsiveness to dopamine agonists in adjuvant therapy or more than 50% reduction of TSH levels in bromocriptine suppression tests were identified in our literature review, the preoperative use of dopamine agonists was not demonstrated in sufficient detail for clinical guidance. In our case, we explored a preoperative 10-day therapy of bromocriptine with a dosage of 2.5 mg every 8 hours to normalize thyroid function, providing a specific regimen for future use of neurosurgeons. However, because TSHomas are rare, data on the preoperative use of dopamine agonists for tumor shrinkage are still lacking. By contrast, successful experiences using preoperative SSAs for restored euthyroidism and tumor shrinkage have been reported in several studies.^[20–22]^ Notably, Yamada et al^[20]^ demonstrated that administration of octreotide prior to surgery could normalize FT4 levels in 83% of patients and shrink tumors in 55% of patients.

**Figure 4:**
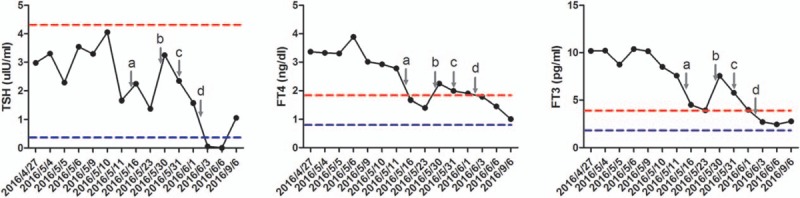
Results of dynamic thyroid function monitoring with medical interventions (gray arrow: (A) start of bromocriptine, (B) start of octreotide and discontinuation of bromocriptine, (C) reuse of bromocriptine and discontinuation of octreotide, (D) pituitary surgery. Dotted line red: upper limit of the normal range, blue: lower limit of the normal range). TSH = thyroid-stimulating hormone, FT3 = free triiodothyronine, FT4 = free thyroxine.

During the preoperative course for the present case, bromocriptine administration was discontinued after thyroid function normalization and was followed by octreotide administration for 4 days because octreotide is recommended for preoperative stabilization of thyroid hormones. However, octreotide therapy resulted in significantly elevated thyroid hormone levels and tachycardia, showing the ineffectiveness of octreotide in our patient. Hormone control was rapidly achieved again after bromocriptine administration was reinstated (Fig. 4). Thus, the combination of negative suppression test results and poor therapeutic response of the patient strongly indicated that our patient was resistant to SSAs. The mechanism of SSA resistance, which has been mainly studied in patients with acromegaly, is complex and is now mostly attributed to the lack of type 2 somatostatin receptor expression and aberrant changes of some emerging novel molecular targets, such as the α subunit of G stimulatory proteins, β-arrestin, fibroblast growth factor receptor 4, aryl hydrocarbon receptor-interacting protein, and tumor suppressor gene *ZAC1*.^[23–26]^ The mechanism of action of SSAs in TSHoma has been considered similar to that in acromegaly showing a positive correlation with expression levels of somatostatin receptors, especially SSTR2 and SSTR5.^[27]^ Interestingly, Chowdhury et al^[28]^ observed that intraoperative occurrence of trigeminocardiac reflex is a negative prognostic factor of postoperative hormonal normalization for pituitary adenomas and hence recommended the intraoperative examination as routine practice. The relationship of trigeminocardiac reflex and medical response warrants further explorations. Investigations examining the resistance of TSHoma to SSAs are limited, but it is hypothesized that the mechanism of this resistance is akin to that in acromegaly.

## 4 Conclusions

Medical treatment, including administration of SSAs with priority and dopamine agonists, is effective in normalizing thyroid function and reducing tumor size. The role of bromocriptine and cabergoline as adjuvant therapy in TSHoma should not be ignored. Moreover, the potential mechanism underlying the therapeutic effects of dopamine agonists in TSHoma treatment warrants further investigation and may lead to the discovery of potential novel therapeutic targets.

## Acknowledgments

The authors express many thanks to the patient for generously authorizing us to share his rare case.

## References

[1] Clarke MJ Erickson D Castro MR Thyroid-stimulating hormone pituitary adenomas. J Neurosurg 2008;109:17–22. 18590428 10.3171/JNS/2008/109/7/0017

[2] Jailer JW Holub DA Remission of Graves’ disease following radiotherapy of a pituitary neoplasm. Am J Med 1960;28:497–500. 14406535 10.1016/0002-9343(60)90181-9

[3] Onnestam L Berinder K Burman P National incidence and prevalence of TSH-secreting pituitary adenomas in Sweden. J Clin Endocrinol Metab 2013;98:626–35. 23295463 10.1210/jc.2012-3362

[4] Sarlis NJ Gourgiotis L Koch CA MR imaging features of thyrotropin-secreting pituitary adenomas at initial presentation. AJR Am J Roentgenol 2003;181:577–82. 12876051 10.2214/ajr.181.2.1810577

[5] Beck-Peccoz P Lania A Beckers A 2013 European thyroid association guidelines for the diagnosis and treatment of thyrotropin-secreting pituitary tumors. Eur Thyroid J 2013;2:76–82. 24783044 10.1159/000351007 PMC3821512

[6] Teramoto A Sanno N Tahara S Pathological study of thyrotropin-secreting pituitary adenoma: plurihormonality and medical treatment. Acta Neuropathol 2004;108:147–53. 15185102 10.1007/s00401-004-0863-x

[7] Rotermund R Riedel N Burkhardt T Surgical treatment and outcome of TSH-producing pituitary adenomas. Acta Neurochir 2017;159:1219–26. 28204898 10.1007/s00701-017-3105-4

[8] Mouton F Faivre-Defrance F Cortet-Rudelli C TSH-secreting adenoma improved with cabergoline. Annales D’endocrinologie 2008;69:244–8. 10.1016/j.ando.2008.02.001 18486933

[9] McLellan AR Connell JM Alexander WD Clinical response of thyrotropin-secreting macroadenoma to bromocriptine and radiotherapy. Acta Endocrinol 1988;119:189–94. 3140550 10.1530/acta.0.1190189

[10] Kuzu F Bayraktaroglu T Zor F G NB A thyrotropin-secreting macroadenoma with positive growth hormone and prolactin immunostaining: a case report and literature review. Nigerian J Clin Pract 2015;18:693–7. 10.4103/1119-3077.158983 26096253

[11] Kienitz T Quinkler M Strasburger CJ Long-term management in five cases of TSH-secreting pituitary adenomas: a single center study and review of the literature. Eur J Endocrinol 2007;157:39–46. 17609400 10.1530/EJE-07-0098

[12] Beckers A Abs R Mahler C Thyrotropin-secreting pituitary adenomas: report of seven cases. J Clin Endocrinol Metab 1991;72:477–83. 1704011 10.1210/jcem-72-2-477

[13] Camacho P Mazzone T Thyrotropin-secreting pituitary adenoma responsive to bromocriptine therapy. Endocr Pract 1999;5:257–60. 15251663 10.4158/EP.5.5.257

[14] Arasho BD Schaller B Sandu N Gender-related differences in pituitary adenomas. Exp Clin Endocrinol Diabetes 2009;117:567–72. 19373750 10.1055/s-0029-1202831

[15] Bevan JS Burke CW Esiri MM Studies of two thyrotrophin-secreting pituitary adenomas: evidence for dopamine receptor deficiency. Clin Endocrinol 1989;31:59–70. 10.1111/j.1365-2265.1989.tb00454.x 2598481

[16] Wood DF Johnston JM Johnston DG Dopamine, the dopamine D2 receptor and pituitary tumours. Clin Endocrinol 1991;35:455–66. 10.1111/j.1365-2265.1991.tb00928.x 1837503

[17] Wang Y Li J Tohti M The expression profile of Dopamine D2 receptor, MGMT and VEGF in different histological subtypes of pituitary adenomas: a study of 197 cases and indications for the medical therapy. J Exp Clin Cancer Res 2014;33:56. 25027022 10.1186/s13046-014-0056-y PMC4223393

[18] Azzalin A Appin CL Schniederjan MJ Comprehensive evaluation of thyrotropinomas: single-center 20-year experience. Pituitary 2016;19:183–93. 26689573 10.1007/s11102-015-0697-7

[19] Malchiodi E Profka E Ferrante E Thyrotropin-secreting pituitary adenomas: outcome of pituitary surgery and irradiation. J Clin Endocrinol Metab 2014;99:2069–76. 24552222 10.1210/jc.2013-4376

[20] Yamada S Fukuhara N Horiguchi K Clinicopathological characteristics and therapeutic outcomes in thyrotropin-secreting pituitary adenomas: a single-center study of 90 cases. J Neurosurg 2014;121:1462–73. 25237847 10.3171/2014.7.JNS1471

[21] van Varsseveld NC Bisschop PH Biermasz NR A long-term follow-up study of eighteen patients with thyrotrophin-secreting pituitary adenomas. Clin Endocrinol (Oxf) 2014;80:395–402. 23848527 10.1111/cen.12290

[22] Socin HV Chanson P Delemer B The changing spectrum of TSH-secreting pituitary adenomas: diagnosis and management in 43 patients. Eur J Endocrinol 2003;148:433–42. 12656664 10.1530/eje.0.1480433

[23] Gadelha MR Kasuki L Korbonits M Novel pathway for somatostatin analogs in patients with acromegaly. Trends Endocrinol Metab 2013;24:238–46. 23270713 10.1016/j.tem.2012.11.007

[24] Gatto F Biermasz NR Feelders RA Low beta-arrestin expression correlates with the responsiveness to long-term somatostatin analog treatment in acromegaly. Eur J Endocrinol 2016;174:651–62. 26888629 10.1530/EJE-15-0391

[25] Ezzat S Wang R Pintilie M FGFR4 polymorphic alleles modulate mitochondrial respiration: a novel target for somatostatin analog action in pituitary tumors. Oncotarget 2017;8:3481–94. 27966451 10.18632/oncotarget.13843 PMC5356897

[26] Efstathiadou ZA Bargiota A Chrisoulidou A Impact of gsp mutations in somatotroph pituitary adenomas on growth hormone response to somatostatin analogs: a meta-analysis. Pituitary 2015;18:861–7. 26115707 10.1007/s11102-015-0662-5

[27] Amlashi FG Tritos NA Thyrotropin-secreting pituitary adenomas: epidemiology, diagnosis, and management. Endocrine 2016;52:427–40. 26792794 10.1007/s12020-016-0863-3

[28] Chowdhury T Nothen C Filis A Functional outcome changes in surgery for pituitary adenomas after intraoperative occurrence of the trigeminocardiac reflex: first description in a retrospective observational study. Medicine 2015;94:e1463. 26376385 10.1097/MD.0000000000001463 PMC4635799

