# Successful management of octreotide-insensitive thyrotropin-secreting pituitary adenoma with bromocriptine and surgery: A case report and literature review: Erratum

**DOI:** 10.1097/MD.0000000000008225

**Published:** 2017-09-29

In the article, “Successful management of octreotide-insensitive thyrotropin-secreting pituitary adenoma with bromocriptine and surgery: A case report and literature review”,^[1]^ which appeared in Volume 96, Issue 36 of *Medicine*, the arrows in panels C and D of Figure 1 were incorrectly placed. The corrected figure is below.

**Figure 1:**
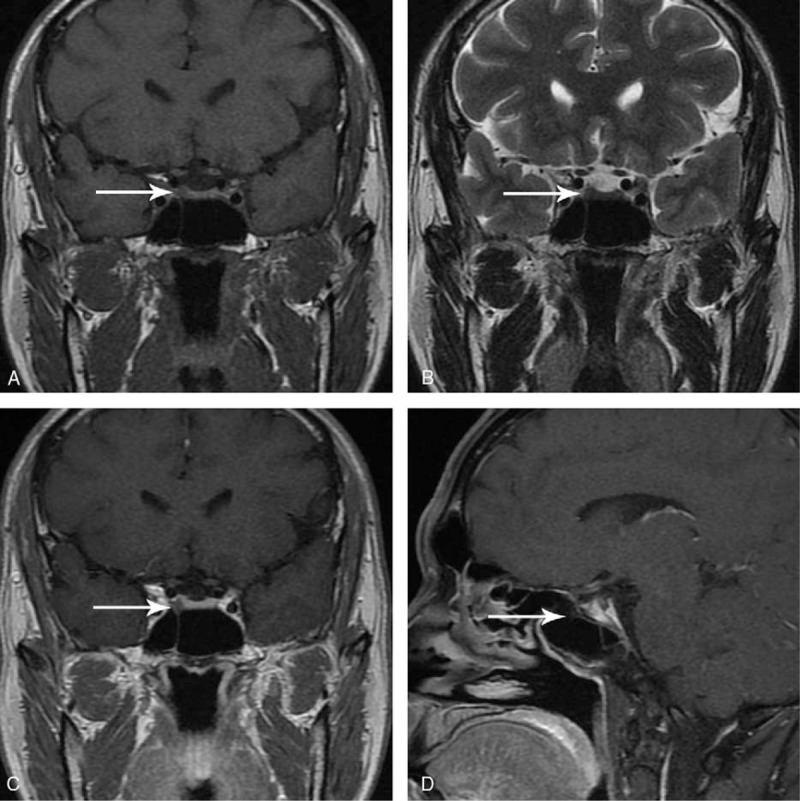
Preoperative MRI characteristics of the pituitary lesion: (A) coronal T1WI, (B) coronal T2WI, (C) coronal-enhanced T1WI, (D) sagittal-enhanced T1WI). MRI = magnetic resonance imaging, T1WI = T1-weighted image, T2WI = T2-weighted image.
